# Alloimmunization in Pregnancy: Patient-reported Quality of Care, Mental Health Effects, and Impact Upon Daily Life

**DOI:** 10.1055/a-2690-9547

**Published:** 2025-09-08

**Authors:** Molly R. Sherwood, Bethany M. Weathersby, Marion E. Granger, Kathryn A. Shanahan, Kara B. Markham

**Affiliations:** 1 Allo Hope Foundation, Tuscaloosa, Alabama; 2 University of South Carolina, Columbia, South Carolina; 3 University of Cincinnati, Cincinnati, Ohio

**Keywords:** fetal hydrops, hemolytic anemia, hemolytic disease, isoimmunization, red blood cell antibodies, red cell antibodies, Rh disease, maternal mental health, high risk pregnancy

## Abstract

**Objective:**

The purpose of this study was to investigate mental health and impacts upon daily life in patients with a history of pregnancy alloimmunization, and secondarily to examine the relationship between disease severity and quality of care on these outcomes.

**Study Design:**

This was a survey administered between November 2022 and February 2023 to U.S. adults with a history of red cell alloimmunization in pregnancy. Mental health outcomes, quality of care, and daily life impacts were reported.

**Results:**

The survey was completed by 127 alloimmunized adults. Anxiety (90.6%), guilt (74.8%), self-doubt (68.0%), isolation (71.4%), depression (68.3%), and symptoms of posttraumatic stress disorder (PTSD) (61.3%) were frequently reported. Mental health support was offered in 24.4%. Respondents reporting a high quality of care in their alloimmunized pregnancies (rating of 8/10 or higher) were significantly less likely than those who perceived receiving lower-quality care to report feelings of guilt ( *p* = 0.0006), self-doubt ( *p* = 0.04), depression ( *p* = 0.03), and symptoms of PTSD ( *p* = 0.001).

**Conclusion:**

Despite the pervasive patient burden, mental health support was infrequently offered, and patients reported low satisfaction with continuity of care and their providers' knowledge of alloimmunization. Clinicians interacting with alloimmunized patients must employ a comprehensive patient-centered approach to address the significant disease burden.

**Key Points:**

- The impact of alloimmunization in pregnancy on patients' mental health and daily living has not been evaluated.
- Females with a history of alloimmunization in pregnancy reported widespread anxiety (115/127; 90.6%), guilt (95/127; 74.8%), self-doubt (85/127; 68.0%), isolation (90/127; 71.4%), depression (86/127; 68.3%), and symptoms of PTSD (76/127; 61.3%); mental health support was offered in 24.4% of the sample (31/127).
- Respondents reporting a high quality of care (rating of 8/10 or higher) were significantly less likely to report feelings of guilt ( *p* = 0.0006), self-doubt ( *p* = 0.04), depression ( *p* = 0.03), and symptoms of PTSD ( *p* = 0.001).
- This study highlights a need to utilize patient-centered comprehensive care models to support this rare high-risk population. Bolstered clinician knowledge and referral to peer support networks, mental health counselors, and social workers may prove highly beneficial to this patient population.

Red cell alloimmunization is a rare complication of pregnancy in which a person of childbearing potential develops red cell antibodies capable of crossing the placenta. These red cell antibodies can cause fetal hemolysis resulting in hemolytic disease of the fetus and newborn (HDFN). If improperly treated, HDFN can result in severe fetal anemia, heart failure, hydrops fetalis, and death. 1 Maternal red cell alloimmunization can occur after exposure to nonself red blood cells through fetomaternal hemorrhage in pregnancy and childbirth, previous blood transfusion, or intravenous drug use. With attentive monitoring and skilled intervention with intrauterine transfusions (IUTs) as indicated, fetal survival can be as high as 98% in highly specialized referral centers, 2 although this can reduce to 80% or lower contingent on country-specific resources. 3

Alloimmunization in pregnancy poses a unique challenge on both clinicians and patients. The clinician manages an unpredictable rare disease, and the patient must rapidly educate themselves to advocate for their children's best chances of survival from HDFN caused at the hand of their own immune system while facilitating care across multiple disciplines, including obstetrics, maternal fetal medicine, neonatology, and hematology. As many patients are sensitized after previous pregnancies, they are likely to have additional children to care for while they manage their high-risk pregnancy and accompanying logistical and financial burdens.

The implications of maternal mental health on maternal morbidity and mortality are widely documented and are currently a subject of significant global scrutiny. 4 5 6 Limited literature discusses the implications of high-risk pregnancy on maternal mental health and aspects of daily life. 7 8 To date, no such investigation has been conducted in the alloimmunized patient population. The objective of this study was to investigate self-reported mental health and impact upon daily life in individuals with a history of alloimmunization in pregnancy. A secondary objective was to examine the impact of disease severity and patient-reported quality of care upon these outcomes.

## Materials and Methods

This investigation was an institutional review board (IRB)-approved retrospective cross-sectional structured survey of people with a history of alloimmunized pregnancy (WIRB-Copernicus Group IRB tracking number: 20224681). Participants were recruited to participate in the survey between November 2022 and February 2023 through the Allo Hope Foundation's (AHF's) social media and private online patient support group. AHF is a U.S. 501(c)(3) nonprofit organization dedicated to serving families, clinicians, and other stakeholders managing alloimmunization and HDFN. Participants were eligible to participate if they were 18 years or older, resided in the United States, and had at least one alloimmunized pregnancy that progressed beyond 12 weeks and completed (i.e., ended in live birth or fetal death). The survey was anonymous, voluntary, and participants were incentivized to participate with a gift card.

Researchers developed the survey utilizing the Checklist for Reporting Results of Interest E-Surveys to reduce risk of bias. 9 The closed web-based survey employed adaptive questioning to eliminate questions that were not applicable to the respondent. Due to the content of the survey, question randomization was not feasible. The questionnaire utilized Qualtrics for administration, automatic data collection, and storage without gathering protected health information. One-time links used cookies to limit access to only one device per link. To preserve anonymity, considering the small patient pool, participants were not asked to report on the location of their treatment. Participants were provided a one-time login link to the survey and directed to provide their email address for incentive payment in a secondary anonymous survey that could not be linked to their survey responses. At the end of the survey, participants were also provided resources for disease information and mental health support, including grief and loss resources.

The survey informed potential respondents of the purpose of the study, length of the survey, data storage processes, investigator contact information, and collected informed consent. They were notified of the risks of participating and their option to withdraw at any time. The full survey is available upon request, but, briefly, it collected comprehensive data regarding participants alloimmunized pregnancies, including severity of disease, management experience, outcomes, and mental health impacts. Participants could respond with “don't know” or “not applicable” if they were uncertain of the answer to the question. Only complete questionnaires were included in analysis; “don't know;” and “not applicable” were considered complete responses but not calculated in analysis for the given question.

For each alloimmunized pregnancy, participants provided information including when they were diagnosed, antibody type, titer, frequency of titer assessment, whether or not the fetus was confirmed antigen positive, and what testing was utilized to determine fetal antigen status. If applicable, frequency of middle cerebral artery (MCA) Doppler scans and the highest multiples of the median values were collected as was antenatal intervention information including use of intravenous immune globulin (IVIG) or plasmapheresis to delay time to IUT, phenobarbital for fetal hepatic maturity, frequency and number of IUTs, and any procedural complications. Delivery timing, neonatal interventions, and outcomes were also probed. Specific management and outcomes are reported elsewhere. 10

In addition to the medical trajectory of their pregnancies, information was obtained regarding daily life impact surrounding travel, impact on work and home life, financial impact, and emotional burden through categorical variables and dichotomous checklists. Five-point Likert satisfaction and agreement scales were offered for various metrics surrounding overall pregnancy care and mental health impact of alloimmunized pregnancies. For the purposes of this study, a response of “somewhat satisfied” or “highly satisfied” was categorized as being satisfied and a response of “somewhat agree” or “highly agree” was categorized as agreement with the statement. Participants were asked to rate the consequences of their alloimmunized pregnancy from 1 to 10 (1 being unburdened, and 10 being the most burdened) related to logistical impact, relational impact, emotional impact, physical impact to themselves, and physical impact to their child, in addition to providing overall ratings for quality of care from 1 (lowest) to 10 (highest).

Respondents were classified as having a history of severe disease if any of their alloimmunized pregnancies had an antigen-positive fetus and required plasmapheresis and/or IVIG, required intervention with IUT prior to 24 weeks, was hydropic, or the pregnancy resulted in fetal or neonatal death. A threshold of 8 out of 10 or above was chosen to define high-quality care based on previous literature demonstrating that the average patient rating of their obstetrician was an 8 out of 10. 11 Other variables related to care quality included satisfaction with the continuity of care across providers and whether they changed doctors to find better care. Participants were asked about mental health outcomes as a result of their alloimmunized pregnancies, including symptoms of anxiety, guilt, self-doubt, isolation, depression and posttraumatic stress disorder (PTSD).

Variables of self-reported mental health and daily life burden were categorized and stratified by presence of severe disease and high quality of care. Descriptive statistics and chi-square or Fisher exact *p* -values were reported ( *p* -values < 0.05 were considered significant). Median (Q1–Q3) was reported when applicable for non-normally distributed data. All analyses were conducted in SAS 9.4.

## Results

Survey links were offered to 172 potential participants, and 130 surveys were initiated (75.6%). A total of 127 of 130 surveys completed all required sections (97.7%). The majority of respondents were non-Hispanic White (91.3%), had bachelor's degrees or higher (61.4%), reported family income at or above $52,200 (81.9%), and carried private insurance (64.6%). Most respondents had two or three living children (42.9 and 29.4%) and reported one or two previous alloimmunized pregnancies (61.4 and 26.0%; Table 1 ).

**Table 1:** Participant demographics and pregnancy history

Demographic and pregnancy characteristics	Participants ( *n* = 127)
	*n* (%)
Race and ethnicity	
Non-Hispanic White	116 (91.3)
Hispanic White	5 (3.9)
Non-Hispanic Black	2 (1.6)
Other	4 (3.1)
Education	
High school or equivalent	12 (9.4)
Some college	17 (13.4)
Associates degree or certificate program	20 (15.7)
Bachelors degree	40 (31.5)
Masters degree	31 (24.4)
Doctoral degree	7 (5.5)
Family income	
Prefer not to respond	3 (2.4)
Less than $52,200	20 (15.7)
$52,200–$156,600	70 (55.1)
More than $156,600	34 (26.8)
Insurance	
Private	82 (64.6)
Government-provided	26 (20.5)
Private and government-provided	3 (2.4)
Military	10 (7.9)
Other	5 (3.9)
None	1 (0.8)
Description of region of residence	
Suburban	59 (46.5)
Urban	41 (32.3)
Rural	27 (21.3)
Number of living children	
1	11 (8.7)
2	54 (42.9)
3	37 (29.4)
4	12 (9.5)
5+	12 (9.5)
Number of completed alloimmunized pregnancies that ended in live birth or fetal death	
1	78 (61.4)
2	33 (26.0)
3	8 (6.3)
4	5 (3.9)
5+	3 (2.4)

The respondents reported on a total of 200 alloimmunized pregnancies. These were grouped as the respondents' collective alloimmunized pregnancy history (e.g., if they received an IUT in one of two alloimmunized pregnancies, they were tabulated as having a history of receiving an IUT). Respondents had a single red cell antibody in 56.7% of cases (72/127) and multiple antibodies in 40.2% (51/127). The majority of respondents had experienced MCA Doppler ultrasounds (111/127; 87.4%). A quarter of respondents had a history of IUT (26.8%, 34/127) and 57.5% (73/127) had a history of neonatal intensive care unit admission for their child. Nearly 60% of respondents reported that their neonates required phototherapy (59.8%,76/127), whereas 28.3% (36/127) and 7.9% (10/127) reporting having a neonate that required a simple transfusion or an exchange transfusion, respectively. A history of fetal death due to HDFN was reported by 7.1% (9/127) respondents. There were no reported cases of neonatal death due to HDFN ( Table 2 ).

**Table 2:** Participants' pregnancy presentation, management, and outcome history across alloimmunized pregnancies

Characteristic	All participants ( *n* = 127)	History of severe disease ( *n* = 22)	No history of severe disease ( *n* = 105)	Chi-square *p* -value
	*n* (%)	*n* (%)	*n* (%)	
Single antibody ( *n* = 72)				
Only anti-D	16 (22.2)	2 (9.1)	14 (13.3)	0.59
Only anti-E	22 (30.6)	0 (0.0)	22 (21.0)	N/A
Only anti-K	23 (31.9)	2 (9.1)	20 (19.0)	0.26
Only anti-c	4 (5.6)	0 (0.0)	4 (3.8)	N/A
Multiple antibodies ( *n* = 51)				
Anti-D + others	28 (54.9)	8 (36.4)	20 (19.0)	0.07
Anti-E + others	21 (41.2)	4 (18.2)	17 (16.2)	0.05
Anti-K + others	9 (17.6)	5 (22.7)	4 (3.8)	0.002
Anti-c + others	15 (29.4)	3 (13.6)	12 (11.4)	0.77
Pregnancy management history				
MCA Doppler ultrasounds	111 (87.4)	21 (95.4)	90 (85.7)	0.21
Intravenous immune globulin	14 (11.0)	13 (59.1)	1 (0.1)	<0.0001
Plasmapheresis	5 (3.9)	5 (22.7)	0 (0.0)	N/A
Phenobarbital	7 (5.5)	5 (22.7)	2 (1.9)	0.001
Intrauterine transfusion	34 (26.8)	20 (90.1)	14 (13.3)	<0.0001
Fetal/neonatal outcomes				
NICU admission	73 (57.5)	20 (90.9)	53 (50.5)	0.0005
Phototherapy	76 (59.8)	18 (81.8)	58 (55.2)	0.02
Top-up transfusion	36 (28.3)	15 (68.2)	21 (20.0)	<0.0001
Exchange transfusion	10 (7.9)	5 (22.7)	5 (4.8)	0.004
Death in utero due to alloimmunization	9 (7.1)	9 (40.9)	0 (0.0)	N/A

Abbreviations: MCA, middle cerebral artery; N/A, not applicable; NICU, neonatal intensive care unit.

Table 3 demonstrates various impacts on daily life imposed by respondents' alloimmunized pregnancy experience. Difficulty coordinating childcare (79/127; 62.2%), missing work more than usual (72/127; 56.7%), and an inability to engage in daily life (73/127; 57.5%) were among the most commonly reported effects. Half of the sample reported making the decision to have less children than initially planned because of their condition (62/127; 49.6%). Participants with a history of severe disease were significantly more likely to report missing work more than usual ( *p* = 0.03), being unable to keep up with home responsibilities ( *p* = 0.01), having difficulty receiving insurance coverage ( *p* = 0.0002), experiencing unpleasant side effects from treatments ( *p* < 0.0001), and changing doctors to find better care ( *p* = 0.04) than participants without a history of severe disease.

**Table 3:** Alloimmunized participants' impacts upon daily life

	All participants ( *n* = 127)	History of severe disease ( *n* = 22)	No history of severe disease ( *n* = 105)	Chi-square *p* -value
Impacts on daily life (yes responses)	*n* (%)	*n* (%)	*n* (%)	
Missed work more than usual	72 (56.7)	17 (77.3)	55 (52.4)	0.03
Difficulty coordinating care for other children	79 (62.2)	16 (72.3)	63 (60.0)	0.26
Unable to engage in daily life due to pregnancy-related distractions and worries	73 (57.5)	14 (63.6)	59 (56.2)	0.52
Undertook a significant financial burden	43 (33.9)	9 (40.9)	34 (32.4)	0.44
Unable to keep up with home responsibilities	51 (40.2)	14 (63.6)	37 (35.2)	0.01
Faced challenges receiving insurance coverage	39 (30.7)	14 (63.6)	25 (23.8)	0.0002
Experienced unpleasant side effects from treatments	29 (22.8)	19 (86.4)	10 (9.5)	<0.0001
Experienced debilitating side effects from treatments	6 (4.7)	6 (27.3)	0 (0.0)	N/A
Changed doctors to find better care	61 (48.0)	15 (68.2)	46 (43.8)	0.04
Decided to have less children than initially planned due to alloimmunization	63 (49.6)	13 (59.1)	50 (47.6)	0.33

Abbreviation: N/A, not applicable.

The median rating for quality of care was 7 out of 10 (10 being the highest quality care) (Q1–Q3 5–9). Participants reported being satisfied with their obstetrician/midwife's knowledge of their condition in 35.7% (45/127), satisfied with their maternal fetal medicine specialist's knowledge in 66.1% (84/127), and satisfied with continuity of care across providers in 42.5% (54/127). Table 4 depicts the mental health impact of participants' alloimmunized pregnancies stratified by history of severe disease and high versus low self-perceived quality of care. Nearly half of respondents reported having changed doctors to find better care (61/127; 48.0%). In the full sample, anxiety (115/127; 90.6%), guilt (95/127; 74.8%), self-doubt (85/127; 68.0%), isolation (90/127; 71.4%), depression (86/127; 68.3%), and symptoms of PTSD (76/127; 61.3%) were frequently reported as a result of respondents' alloimmunized pregnancies. Symptoms of PTSD were reported significantly more often in respondents with a history of severe disease ( *p* = 0.03), but the remaining mental health outcomes were not significantly different between respondents with and without a history of severe disease. In contrast, respondents who reported a high quality of care were significantly less likely than those who received lower-quality care to report feelings of guilt ( *p* = 0.0006), self-doubt ( *p* = 0.04), depression ( *p* = 0.03), and symptoms of PTSD ( *p* = 0.001). Mental health support was offered in 24.4% of the sample (31/127) and was not significantly different between respondents with severe versus nonsevere disease or high versus low quality care.

**Table 4:** Alloimmunized participants' mental health consequences and mental health support

	All participants ( *n* = 127)	History of severe disease ( *n* = 22)		No history of severe disease ( *n* = 105)		Chi-square *p* -Value	Self-reported quality of care <8 ( *n* = 80)		Self-reported quality of care 8+ ( *n* = 47)		Chi-square *p* -value
Experienced new or worsening feelings of (yes responses)	*n* (%)	*n* (%)	Row %	*n* (%)	Row %		*n* (%)	Row %	*n* (%)	Row %	
Anxiety	115 (90.6)	20 (90.9)	17.4	95 (90.5)	82.6	0.9 a	74 (92.5)	64.3	41 (87.2)	35.7	0.3
Guilt	95 (74.8)	17 (77.3)	17.9	78 (74.3)	82.1	0.8	68 (85.0)	71.6	27 (57.4)	28.4	0.0006
Self-doubt	85 (68.0)	15 (68.2)	17.6	70 (68.0)	82.4	1.0	59 (74.7)	69.4	26 (56.5)	30.6	0.04
Isolation	90 (71.4)	18 (81.8)	20.0	72 (69.2)	80.0	0.2	61 (77.2)	67.8	29 (61.7)	32.2	0.06
Depression	86 (68.3)	16 (72.7)	18.6	70 (67.3)	81.4	0.6	60 (75.0)	69.8	26 (56.5)	30.2	0.03
Symptoms of PTSD	76 (61.3)	18 (81.8)	23.7	58 (56.9)	76.3	0.03	57 (72.2)	75.0	19 (42.2)	25.0	0.001
Offered mental health support	31 (24.4)	5 (22.7)	16.1	26 (24.8)	83.9	0.8 a	13 (17.1)	41.9	18 (35.3)	58.1	0.06

Abbreviation: PTSD, posttraumatic stress disorder.

a An unstable *p* -value.

Self-reported burden of disease is reported in Table 5 . Participants' most highly rated burden was the emotional consequence of their alloimmunized pregnancies (median: 8.0; Q1–Q3 5.0–10.0). Mothers with a history of severe disease were significantly more likely to report a high burden from logistical requirements ( *p* = 0.002), and physical consequences to themselves (p ≤ 0.001) and to their baby ( *p* = 0.02). Self-reported quality of care did not have a significant impact on any burden categories.

**Table 5:** Patient disease burden during alloimmunized pregnancy

Self-reported burden rating (1–10)	All participants ( *n* = 127)	History of severe disease ( *n* = 22)	No history of severe disease ( *n* = 105)	Wilcoxon *p* -value	Self-reported quality of care < 8	Self-reported quality of care 8+	Wilcoxon *p* -value
	Median (Q1–Q3)	Median (Q1–Q3)	Median (Q1–Q3)		Median (Q1–Q3)	Median (Q1–Q3)	
Logistical consequences	6.0 (4.0–8.0)	8.0 (7.0–10.0)	5.0 (3.0–7.0)	0.002	6.0 (3.0–8.0)	6.0 (4.0–9.0)	0.45
Relational consequences	4.5 (2.0–6.0)	6.0 (2.0–8.0)	4.0 (2.0–6.0)	0.05	5.0 (2.0–7.0)	4.0 (2.0–6.0)	0.38
Emotional consequences	8.0 (5.0–10.0)	9.5 (6.0–10.0)	8.0 (5.0–10.0)	0.12	8.0 (7.0–10.0)	8.0 (5.0–10.0)	0.18
Physical consequences to self	1.0 (0.0–6.0)	7.0 (3.0–9.0)	1.0 (0.0–4.0)	<0.001	1.0 (0.0–6.0)	2.0 (0.0–6.0)	0.76
Physical consequences to baby	5.0 (1.0–9.0)	9.0 (3.0–10.0)	5.0 (1.0–8.0)	0.02	5.0 (1.0–9.0)	6.0 (1.0–9.0)	0.77

## Discussion

This is the only known investigation of the mental health impact and daily life implications of alloimmunization on affected individuals. Considering that the condition is rare and the severity can vary unpredictably, findings were further analyzed in the context of quality of care and history of severe disease. Respondents experiencing alloimmunized pregnancy reported extremely high rates of negative mental health consequences, largely independent of the severity of their disease (the exception being symptoms of PTSD, a reasonable association as those with severe disease experienced fetal loss and/or the need for invasive procedures). The alloimmunized pregnancy experience frequently and negatively affected respondents' daily living including impact on career, childcare, and ability to engage in daily life. Respondents with a history of severe disease reported a greater physical burden for themselves and their baby, and they were more likely to note unpleasant or debilitating side effects from their treatments. Despite this, mental health support was offered in only 24.4% of the sample. Previous literature demonstrates a broad impact of shock, fear, frustration, grief, isolation, anger, sadness, and guilt in women navigating high-risk pregnancy. 12 High-risk pregnancy is known to impose negative mental health impacts including anxiety and depression on the patient, 7 13 14 although most investigations are limited to an inpatient setting. 15 PTSD in the high-risk pregnant population, though inconsistently evaluated, has been reported at a rate of up to 43%. 16 No studies have examined a comprehensive impact on patients experiencing alloimmunized pregnancy. In the setting of alloimmunization, the mother's diagnosis or disease severity is out of the patient's control and not related to overall health otherwise. Lifestyle changes do not impact the disease trajectory and no outward symptoms give insight into the fetus' health and well-being during the pregnancy. Patients are entirely reliant upon their providers to monitor and intervene appropriately. As such, the patient–provider relationship is particularly critical. Previous investigation proposed that distress alone does not predict satisfaction with care; however, information sharing, communication, and strong relationships between patients and providers do predict satisfaction. 17 This study similarly indicates that higher satisfaction with care was associated with significantly lower rates of self-reported guilt, self-doubt, and depression but not with severity of disease.

Respondents with a history of severe disease may have experienced fetal death and/or invasive and aggressive treatments including IUTs, plasmapheresis, or IVIG. These respondents were more likely to report symptoms of PTSD than those without a history of severe disease and yet were no more likely to be offered mental health support. They also reported a more significant physical burden to themselves and their baby and were likely to report unpleasant or debilitating side effects from their treatments. In this sample, 17% of respondents had a history of severe disease and 27% had previously received an IUT, similar to previous literature demonstrating that approximately 22 to 23% of antigen-positive fetuses with critical titers require IUT. 18 19 Predictors of severe disease are illusive, 20 and severe disease can manifest quickly. 21 Centers managing alloimmunized pregnancies must consider that any pregnancy with an antigen-positive fetus can progress to severe HDFN and anticipate a need for comprehensive support.

Clinicians should assume that their alloimmunized patient is experiencing a significant mental health burden and is managing widespread negative impacts on her daily life during her pregnancy, even in mild cases. Referral to a mental health specialist should be offered and screening for anxiety, depression, and PTSD is especially critical in this population during pregnancy and after birth. Considering the challenges these patients face with career, childcare, and activities of daily living, referral to social work or resource centers within the hospital system may be necessary.

Alloimmunized patients may receive care from obstetrics, maternal fetal medicine, neonatology, pediatrics, and hematology throughout the management course, and patients in this study report infrequent satisfaction with continuity of care. Peripartum care coordination models may help to alleviate this burden and have been utilized to address PTSD, stress, anxiety, depression, and patient satisfaction. 22 23 24 While these models have been shown to be well received by clinicians, remaining challenges include training and staffing. 25 26 27 A centralized virtual program may help to reduce these burdens, especially considering the rarity of the disease.

Social support is crucial to addressing reported feelings of isolation and guilt. 28 Because of the complexity and rarity of alloimmunization, patients often seek support from online patient groups in the absence of knowing people with similar lived experience in their daily lives. Clinicians may consider referring alloimmunized patients to the Allo Hope Foundation for peer support, educational tools, and individual counsel.

Considering the lack of available patient data at any one clinical center, most existing literature on this disease leans heavily on retrospective chart review evaluations. 3 19 29 Mental health outcomes are not typically evaluable in these settings as screening is not consistently implemented. Beyond convenience sampling, although sometimes a necessity in rare disease research, 30 future research on this topic should include prospective multicenter assessment to systematically evaluate mental health impact at the time of pregnancy. Additional examinations could explore the effect of peer support models or care coordination strategies in this setting.

Some limitations should be considered. The participants in this study were recruited from a patient-advocacy organization-led support group. Respondents were likely to be White with advanced education and may not represent minority or low-socioeconomic status patients. It is possible that these results reflect a more favorable outlook on these metrics of well-being as the participants in this study had the advantage of having online peer support and education on their condition.

One limitation of this investigation is that it is based on respondent recall of the impacts of their previous pregnancies. Because this is recall-based, it was not appropriate to administer standardized tools to diagnose mental health conditions, and previous investigations have called into question the diagnostic accuracy of mental health screening tools in high-risk pregnancy. 31 Results should be interpreted as a reflection of the patients' overall self-reported well-being rather than a definitive clinical diagnosis of mental health disorders.

This is the only known investigation of the comprehensive impact of alloimmunization on the affected patient. In the absence of prospective multicenter investigations in this elusive population, which would require years of evaluation even when conducted across many sites, clinicians must make decisions about how to best support their patients based on the available evidence. This study is the first of its kind to shed light on the need for comprehensive support in this population and the value of high-quality care on patients' well-being, independent of disease severity.

This investigation highlights a need for improved mental health screening, referral, and trust between patient and provider to improve patient-reported quality of care. Clinicians supporting patients during alloimmunized pregnancies must not only offer skilled monitoring and prompt treatment, but also address unmet needs in the patients' overall well-being. In patients whose alloimmunization progresses to severe disease, clinicians must anticipate the patient's physical burdens and side effects in addition to their increased likelihood of ensuing PTSD, which can manifest over time after the pregnancy ends. 16 In addition to improving education around disease management, clinicians may consider referring patients to patient support groups, and to licensed mental health workers and social workers for comprehensive support.
